# Fastball pitching performance only slightly decreases after mobility impediment of the pelvis and trunk—Do (catch-up) compensation strategies come into play?

**DOI:** 10.3389/fspor.2022.1044616

**Published:** 2022-11-23

**Authors:** A. J. R. Leenen, Bart van Trigt, M. J. M. Hoozemans, H. E. J. Veeger

**Affiliations:** ^1^Department of Human Movement Sciences, Amsterdam Movement Sciences, Vrije Universiteit Amsterdam, Amsterdam, Netherlands; ^2^Department of Biomechanical Engineering, Delft University of Technology, Delft, Netherlands

**Keywords:** baseball, kinematic chain, catch-up phenomenon, biomechanics, performance, injury, shoulder, elbow

## Abstract

**Background:**

Baseball pitching performance can be mechanically explained by the summation of speed principle and the principle of optimal coordination of partial momenta. Impeding optimal energy generation or transfer by or between the pelvis and trunk segments could provide valuable insight into possible compensation or catch-up mechanisms that may manifest themselves based on these principles.

**Aim:**

The aim of the present study was to explore the effects of experimentally impeding the mobility of and between the pelvis and trunk segments (1) on ball speed and mechanical peak joint power, and (2) on mechanical peak load of the elbow and shoulder joints at maximal external rotation (MER) during fastball pitching.

**Methods:**

Eleven elite baseball pitchers (mean age 17.4, SD 2.2 years; mean pitching experience 8.9, SD 3.0 years) were instructed to throw at least 15 fastballs as fast and accurately as possible under two conditions. One condition involved impeding the mobility of the pelvis and trunk segments to hamper their ability to rotate independently, which consequently should affect the separation time, defined as the time interval between the pelvis and trunk peak angular velocities. In the other condition, pitchers threw unimpeded. Ball speed, mechanical peak joint power and peak net moment of the elbow and shoulder at MER were compared between conditions using Generalized Estimating Equations (GEE).

**Results:**

In the impeded pitching condition, the mean difference of the separation time was 12.4 milliseconds [95% CI (4.0, 20.7)] and for ball speed 0.6 mph [95% CI (0.2, 0.9)] lower compared to the unimpeded condition. Only the peak pelvic angular velocity, in addition to the trunk, upper arm and forearm, was 45 deg/s [95% CI (24, 66)] higher impeded condition. The mean differences of the joint power and net moments at the shoulder and elbow did not reach statistical significance.

**Conclusion:**

In elite adolescent baseball, the observed pitching performance after experimentally impeding pelvic and trunk mobility undermines a potential distal catch-up strategy based on the summation of speed principle. The increased peak pelvic angular velocity may indicate a compensation strategy following the optimal coordination of partial momenta principle to practically maintain pitching performance.

## Introduction

One of the most remarkable features of baseball pitching is the ability to reach high end-point velocities, with ball speeds up to 100 miles per hour. How baseball players pull this off has been subject of much biomechanical research (1, 2). The most well-known biomechanical principles that probably underlie the mechanics to explain baseball pitching performance are the summation of speed principle (also coined as the “kinetic chain”) and the principle of optimal coordination of partial momenta (3–5). The principle of optimal coordination of partial momenta considers the human body as a linked segment model and states that to achieve high end-point velocities at the most distal segment (i.e., the hand) all segments must reach their peak segment angular velocity at the same time (3, 4). Separation times, defined as the time intervals between peak angular velocities of two adjacent segments (6–8), are expected to be around zero according to this principle. The summation of speed principle, which also considers the human body as a linked segment model, does not require peak segment angular velocities to occur at the same time, but is based on a proximal-to-distal sequence in the rotations of body segments (3, 9). This principle states that maximized end-point velocity at the most distal segment can be achieved when a succeeding distal segment begins to accelerate its rotation when the preceding proximal segment reaches its highest peak angular velocity, and at best achieves a higher peak angular velocity than the preceding segments (3, 9). This requires highly coordinated whole-body movements in which kinetic energy is generated, conserved and transferred from proximal body segment(s) to distal body segment(s), which is often referred to as the kinetic or kinematic chain (9, 10). According to this principle, separation times between peak angular velocities of two adjacent segments are not expected to be zero but are likely to be positive, assuming that the proximal-to-distal sequence is followed, around an optimum suitable for achieving maximum end-point velocity. As stated by Putnam (3), some striking movements, such as the forehand stroke in tennis, follow the principle of optimal coordination of partial momenta. However, most throwing or striking movements, such as the baseball pitch, are believed to be more likely to follow the summation of speed principle and demonstrate the proximal-to-distal sequence (3, 4). In fact, empirical evidence has been provided to support that baseball pitching follows the summation of speed principle (1, 6). These studies focused on the pelvis and trunk peak angular velocities and showed that the separation time, defined as the time interval between the peak angular velocities of the pelvis and trunk segments, was positively associated with ball speed during baseball pitching (1, 6). In baseball pitching, however, the partial momenta theory has not been subject of research.

Regardless of the existence of the summation of speed or optimal coordination of partial momenta principle, the high end-point velocities observed in baseball pitching depend on the contribution of all segments in the chain. Breaking an (early) link in the chain, for instance due to fatigue, could however affect performance and end-point velocity in baseball pitching differently according to the two principles. Following the summation of speed principle, breaking an early link in the chain hampers the process of generation, conservation and transfer of kinetic energy throughout the chain, preventing kinetic energy from being optimally transferred to the hand (11–13). Likewise, in the scenario of the optimal coordination of partial momenta principle, a lower contribution of a link in the chain also hampers the process of total energy generation.

The consequence of the loss of energy production (early) in the chain may manifest itself differently based on the predominant principle underlying baseball pitching, also depending on compensatory mechanisms to maintain pitching performance. A scenario, free of compensatory mechanisms, that applies to both principals, would be that an impeded link in the chain causes less total kinetic energy to arrive at the hand. The total kinetic energy that can be transferred by the hand to the ball is directly related to ball speed (14), meaning that pitching performance also decreases proportionately. In a scenario that considers compensatory mechanisms, the adjustments needed to maintain pitching performance will be different for both principles. In a compensation scenario that is mainly based on the summation of speed principle, the loss of kinetic energy due to an impediment in a preceding segment is compensated for by the successive segments. As a result of this compensation mechanism, the same amount of kinetic energy is transferred to the hand as compared to the situation in which the kinetic chain is not impeded and pitching performance is maintained. This compensation mechanism is referred to as the “catch-up” phenomenon (13). In a compensation scenario that is mainly based on the principle of optimal coordination of partial moments, the loss of kinetic energy must be compensated for throughout the entire system to ensure that the same total amount of kinetic energy arrives at the most distal segment for pitching performance to be maintained. However, these scenarios are undoubtedly not so clear-cut, and in practice baseball pitchers presumably tend to use both principles combined, thus in the scenario of an impeded link in the chain combined effects can be expected.

Since slightly more than half of the total kinetic energy is generated by the segments of the lower extremity, pelvis and trunk, these segments can be considered as the energy generators of the kinetic chain (14, 15). Considering the importance of these two core segments, modifying this link in the chain by impeding the possibility for optimal energy generation or transfer by or between these two segments might provide insight in the roles of the summation of speed principle or the principle of optimal coordination of partial momenta. In the scenario of the summation of speed principle, and assuming that a compensatory mechanism as the catch-up phenomenon exists, the loss of kinetic energy must be compensated for later in the kinetic chain if performance is to be maintained. In baseball pitching, an impediment in the kinetic chain at the pelvis and trunk is expected to result in increased power production later in the chain to maintain ball speed. According to Kibler (16), a reduction of kinetic energy generated by the pelvis and trunk segments by 20% needs to be compensated for by increasing the angular velocity of the upper arm by 34% to deliver the same amount of total kinetic energy to the hand (16). This compensatory strategy of the catch-up phenomenon is thought to increase the mechanical load on the more distal located joints of the kinetic chain. This in turn may put these joints at an increased risk of developing (overuse) injuries, especially the elbow and shoulder (12, 13, 17). However, to the best of our knowledge, the theory of the catch-up phenomenon has never been experimentally demonstrated in fastball pitching in baseball. In the scenario of the optimal coordination of partial momenta principle, the entire chain has to compensate for the loss of kinetic energy at the pelvis and trunk segments to generate in total the same amount of energy if pitching performance has to be maintained. An impediment at the level of the pelvis and trunk is therefore expected to result in an overall increased power production caused by the entire system to maintain ball speed. However, the question that remains unanswered is whether an impediment at the pelvis and trunk (1) actually leads to compensation mechanisms to maintain performance revealed by changes in mechanical power, and (2) leads to increased mechanical load, especially on the elbow and shoulder.

Given the phases of the baseball pitch (18) and the key events of the pitch that follow pelvis and trunk rotation, maximum external rotation (MER) of the throwing arm is the most critical moment before ball release for both the elbow and shoulder in terms of mechanical load (19). As for the mechanical power, peak power is to be expected toward the end of the cocking phase just before the throwing arm reaches MER (18). The purpose of the present study was therefore 2-fold: to explore the effects of experimentally impeding the mobility of and between segments at the pelvis and trunk level (1) on ball speed and mechanical peak joint power, and (2) on mechanical peak load of the elbow and shoulder joints at MER during fastball pitching in baseball.

## Materials and methods

### Participants

A total of 11 elite baseball pitchers of the Dutch AAA team aged 15–23 years participated in this experimental study (mean age 17.4, SD 2.2 years; mean pitching experience 8.9, SD 3.0 years; mean body mass 80.6, SD 11.7 kilograms; mean body height 1.87, SD 0.06 meters). These pitchers are the best pitchers of their age group in The Netherlands. Participants had to be free from an ongoing injury, pain, or muscle soreness that prevented them from throwing a fastball as they would normally do, as well as free from previous injuries that might lead to restrictions in the kinetic chain (such as permanent restrictions of the range of motion due to for instance surgery). The Faculty of Behavioural and Movement Sciences' local ethics committee approved the study under the reference number VCWE-2019-033, and all participants (or, when relevant, their legal caregiver) gave their written consent according to university policy, after being fully informed about the content and purpose of the study.

### Procedures

**Figure 1:**
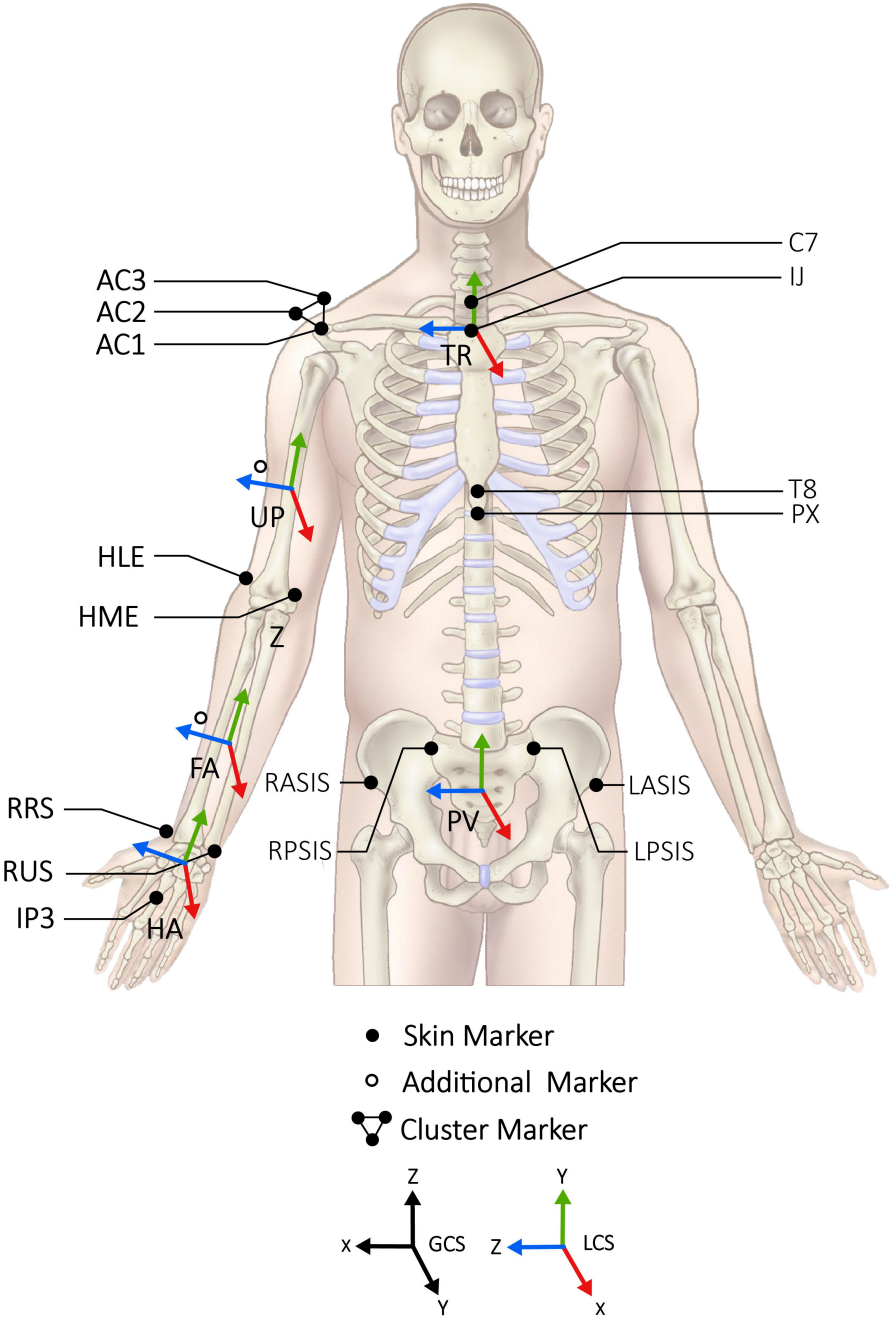
The anatomical landmarks for the reflective markers, the local coordination systems of the pelvis (PV), trunk (TR), upperarm (UP), forearm (FA) and hand (HA) and the definition of the global coordination system are shown.

Data collection was performed in an indoor movement laboratory at the Royal Netherlands Football Association (KNVB), equipped with artificial grass on a custom-made pitching mound and an optoelectronic motion capture area of 5 × 5 m. One side (5-m width) of the laboratory could be opened to the outside, such that participants were able to throw fastballs from the motion capture area into the field toward a rectangular strike zone (height 0.64 m; width 0.38 m) positioned at 0.55 m above the ground and taped on a tensioned net at the regular game distance of 18.4 m (60.5 ft). The participants changed into tight-fitting trousers or shorts and indoor shoes, whereafter 43 reflective markers (10 mm in diameter) were directly attached to anatomic bony landmarks with double-sided tape (Figure 1). These markers were attached according to a modified plug-in-gait model with additional markers on both arms to ensure reliable automatic labelling (20) (Figure 1). Since the plug-in-gait model assumes that the lower extremities are included, markers were only applied for inclusion as these bony landmarks were not used for further analysis. Once the markers were placed, participants were given unrestricted time to warm up as they would normally do for a bullpen, which consisted of running, stretching and a specific throwing session, followed by 5–10 submaximal fastball pitches prior to each condition to become comfortable with the experimental setup.

**Figure 2:**
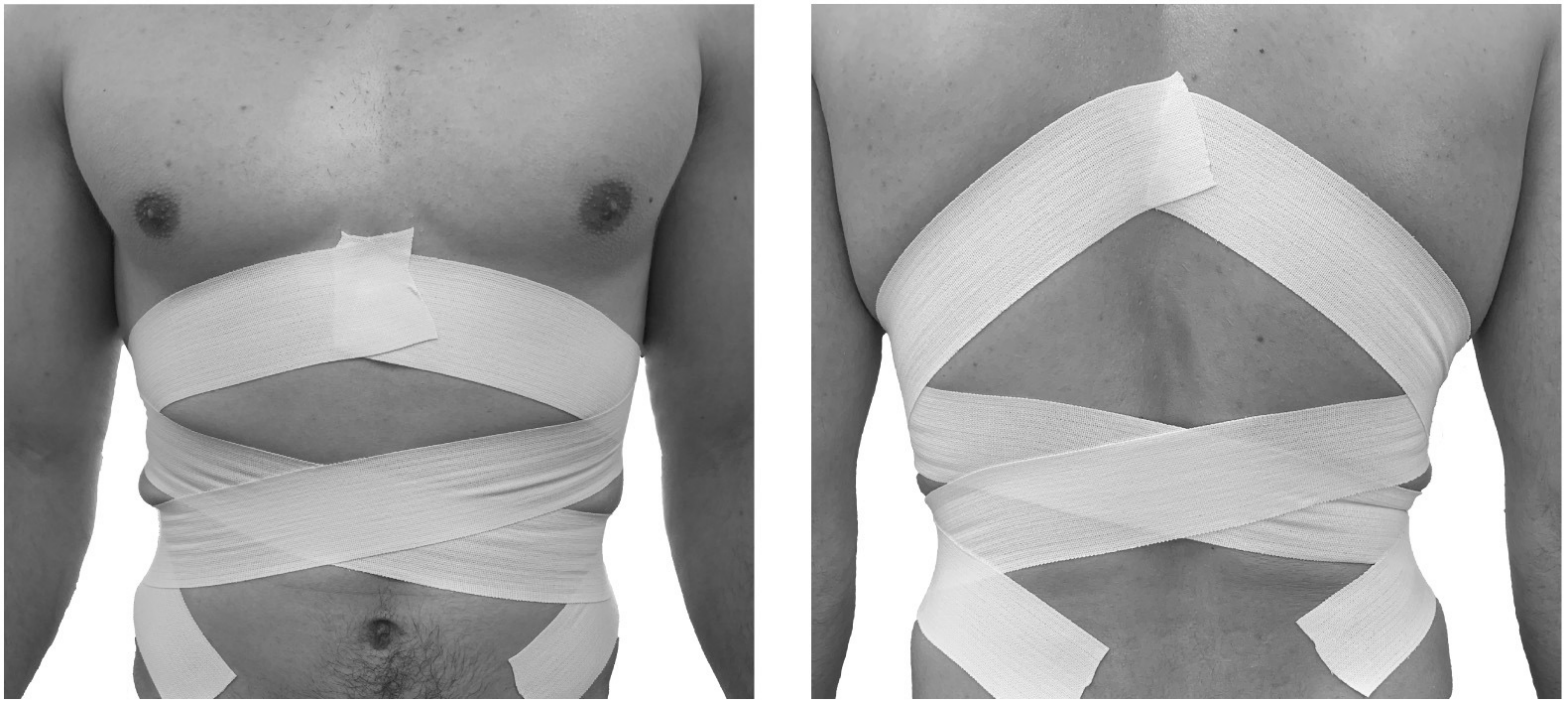
The application of four strips of short-stretch tape in a clockwise and counter clockwise circular fashion to impede the pelvic and trunk mobility.

The current experimental study was set up with two within-subject conditions. One condition acted as a control condition where the pitchers were unimpeded and requested to pitch as they normally would. The other condition involved a mobility impediment at the pelvic and trunk level to impede these segments from their ability to rotate independently, which consequently should affect the influence the time interval between the peak angular velocities of the pelvis and trunk (e.g., separation time). This was accomplished by applying two strips of short stretch tape directly to the skin in a clockwise and counterclockwise circular fashion from the xiphoid process to the anterior superior iliac spine, followed by two strips that were applied from the inferior angle of the scapulae to the posterior superior iliac spine (Figure 2). The sequence of the two conditions presented to each participant was randomly balanced between participants. For both conditions, pitchers were instructed to throw fastballs from a wind-up into the strike zone as fast and accurately as possible. Pitches were considered qualified for the analysis if the backstop was hit. Each participant continued to throw fastballs until a minimum of 25 qualified pitches were captured and all qualified pitches were used for data analysis.

### Data acquisition

Kinematic data were captured using eight optoelectronic motion cameras (Vicon V5 cameras; Vicon Motion Systems Ltd., Oxford, UK) with the Vicon Nexus automatic digitization software (version 2.7.1, Vicon Motion Systems Ltd., Oxford, UK). The cameras were mounted on the walls of the laboratory at an approximate height of 2.3 m around the testing area. The 3D marker positions were captured at 400 frames/s. A radar gun (model Stalker Pro II Sport; Applied Concepts Inc., Plano, TX) was used to capture the ball velocity of each throw directly before the rectangular strike zone was hit.

### Data analysis

Raw marker trajectory data obtained by the optoelectronic motion cameras were exported from Vicon Nexus to PyCharm 2021.1.1 (JetBrains s.r.o., Prague, Czech Republic) running the Python language version 3.8.3. (21) for further data processing and reductions. Captured marker trajectories were excluded from further analysis when participants slipped down the pitching mound and when reflective markers were released from the participants. Gaps in the raw marker trajectories were filled by interpolation using a third-order cubic polynomial and subsequently filtered using a low-pass fourth-order zero-lag Butterworth filter with a cut-off frequency of 12.5 Hz to reduce the effects of sampling error. The local coordinate systems of the pelvis, trunk, upper arm, forearm and hand segments along with the shoulder and elbow joints were defined according to the recommendations of the International Society of Biomechanics (ISB) (22, 23). The global coordinate system was defined with the positive x-axis pointing rightwards, y-axis pointing forward in the throwing direction, and the z-axis pointing upwards, according to the right-hand rule (Figure 1). The instance of maximum external rotation (MER) of the upper arm in the abduction-external rotation position was defined as the first frame in time when the upper arm reached its maximum external rotation position. The segment angular velocities were directly calculated from the rotation matrices as described in Zatsiorski (24). The angular velocity magnitude was calculated as the Euclidean norm using the three components of the angular velocity vector (24). The exact occurrences of the peak angular velocities were analytically determined by fitting a second-order polynomial function using 11 measured data points that consisted of five data samples before and after the samples closest to the maximum angular velocity. The separation time was subsequently calculated as the time interval between the peak angular velocities of the pelvis and the trunk (6–8).

The net forces and moments acting on the shoulder and elbow joints were calculated in the global coordinate system, where the forces and moments of the proximal segment that act on the distal segment, using a top-down inverse dynamic analysis based on the Newton-Euler equation of motions (25). The Euclidean norm of the net moments was calculated to subsequently extract the peak net moment around the instant that the upper arm reaches MER in the abduction-external rotation position. The segment masses, centers of mass locations, and moments of inertia in the coronal and sagittal planes of the upper arm, forearm and hand were estimated using the method developed by Zatsiorsky (26). The moment of inertia about the longitudinal axis of each segment was considered negligible.

The mechanical joint power was calculated directly using the net moments acting on the shoulder and elbow joints and the angular velocities prevailing at the joint over which the mechanical joint power was calculated (27). The mechanical peak joint power was similar to the peak net moments extracted around the moment the upper arm reached the MER in the abduction-external rotation position.

### Statistical analysis

R (R Core Team, version 4.1.1, 2021, Vienna, Austria) was used to perform the statistical analyses, especially by use of the Generalized Estimating Equations (GEE) analysis with the R package “gee” (version 4.13-20) (28, 29). The R packages “ggplot2” (version 3.3.5) and “ggeffects” (version 1.1.1) were used to design the graphs (28, 30, 31). GEE analysis using the exchangeable working correlation structure was used to explore whether the impeded pitching condition, demonstrated statistically significant differences compared to the control condition that consisted of unimpeded pitching. Since the data were not normally distributed, the GEE analysis was used because this analysis yields valid standard errors of the parameter estimates regardless of the distribution. Furthermore, the participants were considered as a random factor to account for the dependency between the repeated pitches within participants. The conditions (unimpeded pitching vs. impeded pitching) were added to the model as categorical predictors (factors), ball speed (mph), separation time (ms), peak angular velocities (°/s), net peak moments (Nm) and peak joint power (W) were the continuous outcome variables. The analyses for the net peak moments and peak joint power were performed separately for the elbow and shoulder. The *post-hoc* analysis for the peak angular velocities was also performed separately for the pelvis, trunk, upper arm and forearm segments. The predictors' regression coefficients (b1) and their corresponding 95% confidence intervals (CI) were determined using the robust covariance matrix estimator (32). An a-priori α level of 0.05 was used to determine statistical significance, but due to multiplicity, the Bonferroni-Holm method was used to control the family-wise error rate by adjusting the rejection criteria for each of the hypotheses tested (33, 34).

## Results

Overall, 240 pitches for the impeded pitching condition and 241 pitches for the unimpeded (normal) pitching condition were analyzed from 11 participants, averaging ~44 pitches per pitcher thrown. In total 69 pitches were excluded from the analysis due to missing indispensable markers from the throwing arm.

**Figure 3:**
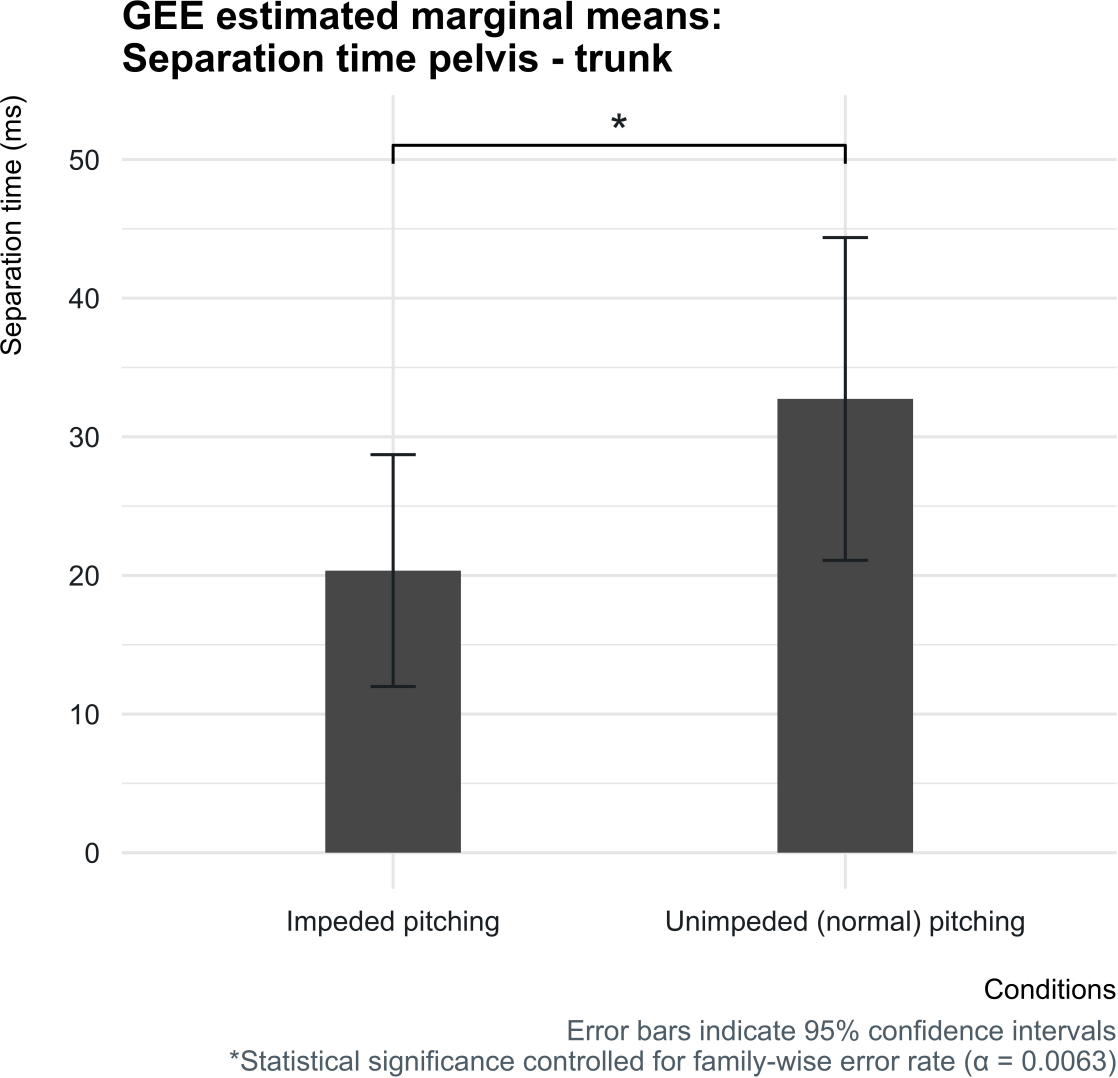
Estimated marginal means for the separation time for both conditions with corresponding 95% confidence interval error bars.

The separation time between the pelvis and trunk was found to be significantly different between both conditions (Figure 3). In the unimpeded pitching condition, the estimated mean separation time between the peak angular velocities of the pelvis and trunk was 32.7 milliseconds [95% CI (21.1, 44.4)], which was 12.4 milliseconds [95% CI (4.0, 20.7)] more compared to the condition in which baseball pitchers had to throw fastballs with impeded pelvic and trunk mobility caused by the applied short-stretchable tape.

**Figure 4:**
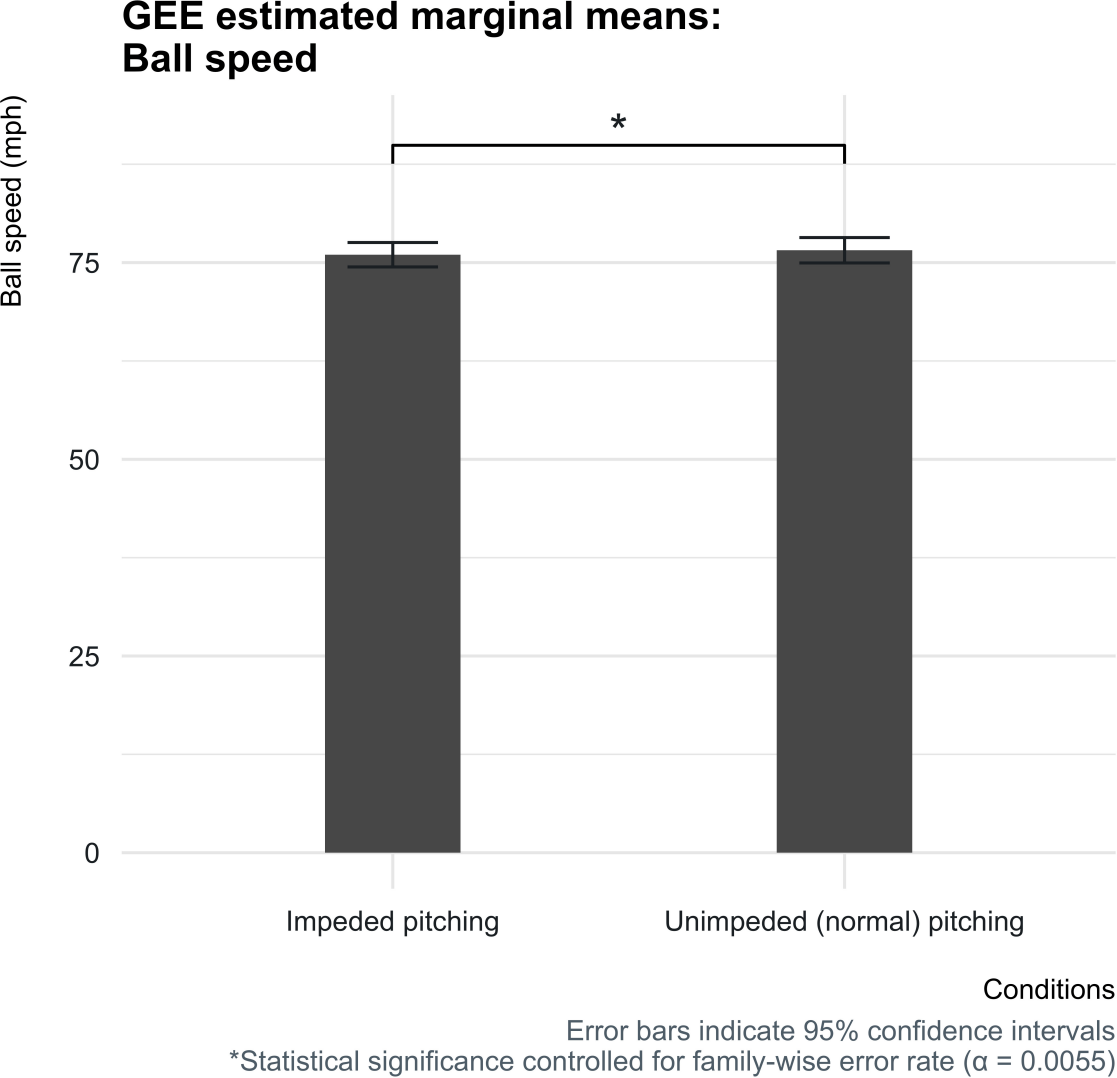
Estimated marginal means for the ball speed for both conditions with corresponding 95% confidence interval error bars.

**Figure 5:**
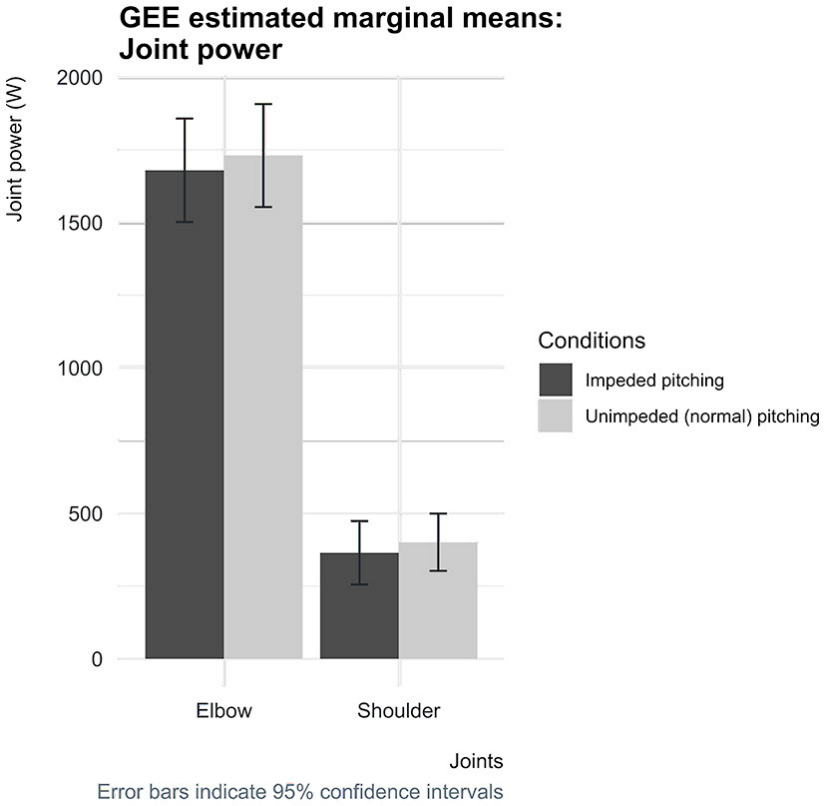
Estimated marginal means for the peak joint power across the shoulder and elbow for both conditions with corresponding 95% confidence interval error bars.

The estimated mean ball speed was 76.0 mph (34.0 m/s) [95% CI (74.4, 77.6)] when baseball pitchers' pelvic and trunk mobility were impeded by the short-stretchable tape, which was significantly lower by 0.6 mph (0.3 m/s) [95% CI (0.2, 0.9)] compared to the normal condition (Figure 4). In addition, when the pitchers' core segments' mobility was impeded, the elbow peak joint power was found to be slightly lower by 51 W [95% CI (5, 98)] and at the shoulder by 36 W [95% CI: (−26, 98)] (Figure 5). These estimated mean peak joint power differences of the elbow and shoulder between the two conditions, however, did not reach statistical significance.

**Figure 6:**
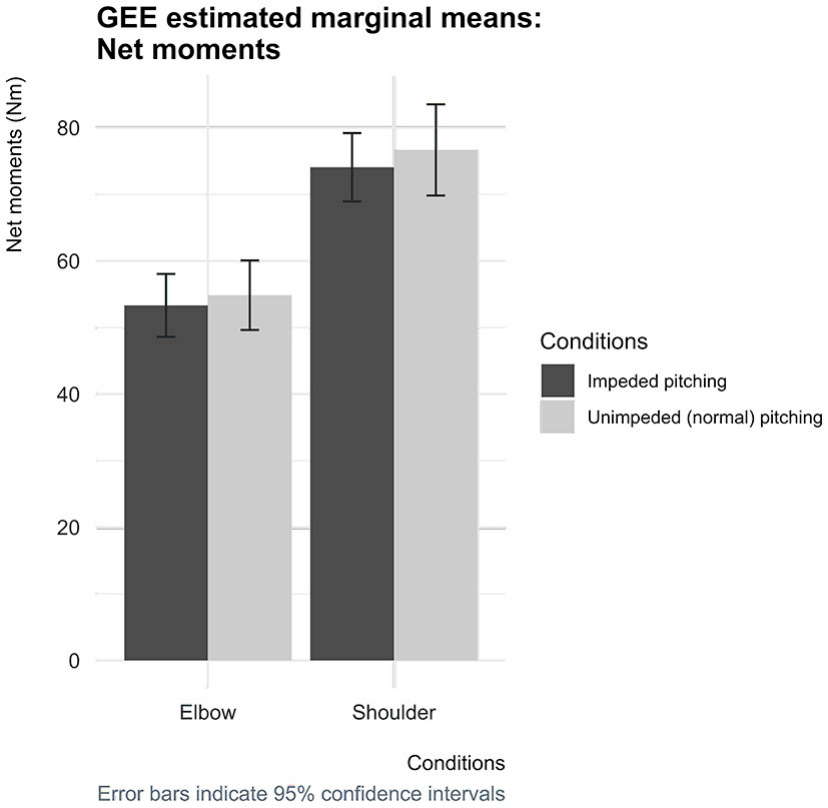
Estimated marginal means for the peak net moments at the shoulder and elbow for both conditions with corresponding 95% confidence interval error bars.

In the condition wherein the pitchers' pelvic and trunk mobility were impeded, the estimated net moment at the elbow was 53.9 Nm [95% CI (49.6, 60.1)] and 74.1 Nm [95% Wald CI (68.9, 79.2)] for the shoulder, which was found to be slightly lower at the elbow by 0.9 Nm [95% Wald CI (−0.2, 2.1)] and by 1.8 Nm [95% CI (−0.7, 4.3)] at the shoulder compared to the normal pitching condition (Figure 6). The estimated mean net shoulder and elbow moments between the two conditions did also not reach statistical significance.

**Figure 7:**
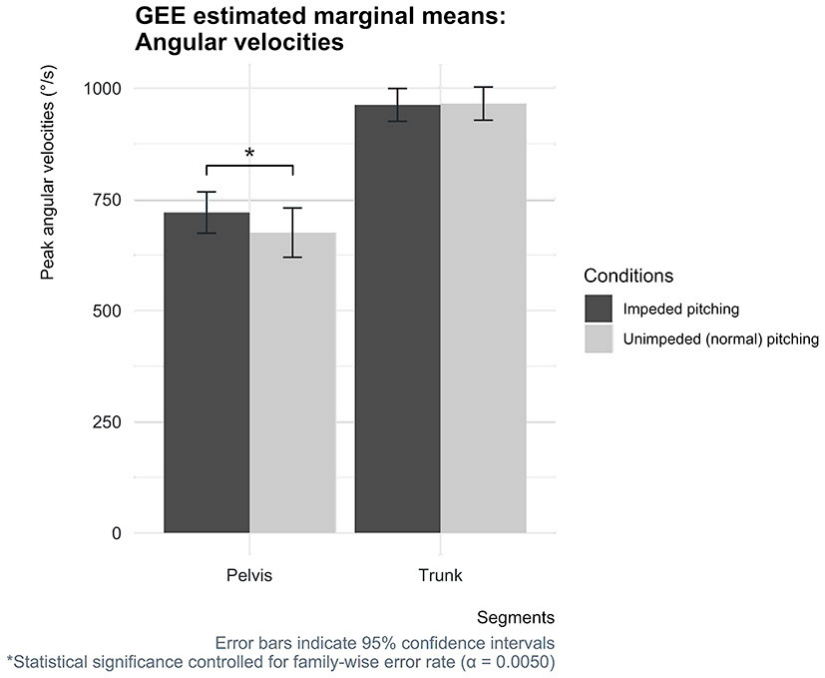
Estimated marginal means for the peak angular velocities of the pelvis and trunk for both conditions with corresponding 95% confidence interval error bars.

**Figure 8:**
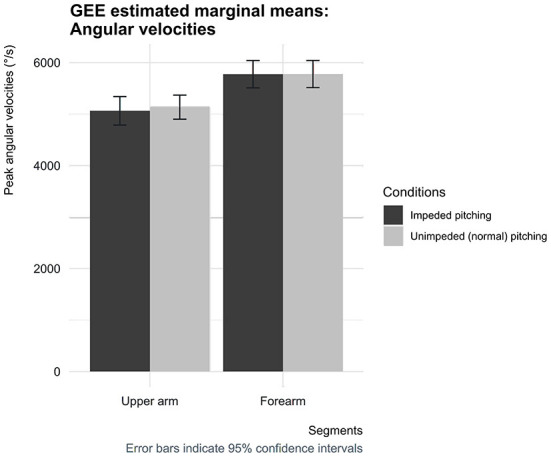
Estimated marginal means for the peak angular velocities of the upper arm and forearm for both conditions with corresponding 95% confidence interval error bars.

The peak pelvic angular velocity differences between the two conditions reached statistical significance, while the peak angular velocity of the trunk, upper arm, and forearm did not. The pelvic peak angular velocity in the impeded condition was 720 deg/s [95% CI (674, 767)], which was 45 deg/s [95% CI (24, 66)] higher compared to the condition in which pitchers could normally throw fastballs [675 deg/s, 95% CI (620, 731)] (Figures 7, 8).

## Discussion

The aim of the current study was 2-fold. The first aim was to examine the effects of experimentally impeding the mobility at the pelvic and trunk level to limit the possibility to contribute to the end-speed through the pelvic-lumbar link, on ball speed and peak joint power during fastball pitching. The successfully impeded pelvis-trunk mobility due to the applied circularly short-stretchable tape caused pitchers to throw fastballs at slightly slower ball speeds. In addition, apparently slightly lower elbow and shoulder joint power was observed, which did not reach statistical significancy. The second aim was to examine the effects of experimentally impeding the mobility of these core segments on elbow and shoulder loading during fastball pitching. The results showed that the elbow and shoulder net moments appeared also slightly lower in the condition where the mobility between the pelvis and trunk was successfully impeded, although these differences were also not statistically significant. Finally, the *post-hoc* analysis revealed that of the predictor variables under study only the peak pelvic angular velocity was found to be higher in the impeded mobility condition compared to unimpeded pitching.

Coupling the pelvis and trunk by the applied circularly short-stretchable tape, making them less capable of rotating independently, was intended to complicate the generation, conservation and transfer of kinetic energy over the pelvic-lumbar link. The always positive—and not zero—separation time was found to be less when pitchers were impeded by the applied circularly short-stretchable tape. The caveat to be made is that regardless of the impediment of the pelvis-lumbar link, pitchers are still able to follow an appropriate proximal-to-distal sequence according to the summation of speed principle. Nevertheless, the impeded mobility of the pelvis and trunk accompanied by less separation time is in line with an impediment of an early link following the summation of speed principle. The seemingly effective method of limiting the possibility to perform optimal following the summation of speed principle, resulted in pitchers throwing at somewhat lower ball speeds. The decreased pitching performance was also accompanied with lower peak joint power at the elbow and shoulder. This means that the impeded pelvic-lumbar link was not compensated for by the segments of the throwing arm, indicating that no compensation mechanisms were used to maintain pitching performance. These findings suggest that compensation mechanisms such as the “catch-up” phenomenon did not manifest itself in pitchers whose pelvis and trunk mobility was impeded by the circularly short-stretchable tape. Besides, as often reported in the literature, these findings fit the assumption that the throwing arm does indeed primarily acts as a funnel to transfer the conserved and generated kinetic energy earlier in the chain to the ball, contributing only marginally to the total generated kinetic energy (14, 15, 18, 35). This is supported, for instance, by the study performed by Howenstein et al. (14) which demonstrated that pitchers, mainly during the arm cocking phase, transferred 177 ± 54 Joules of kinetic energy across the shoulder to the humerus and 155 ± 49 Joules across the elbow to the forearm, while the generation of kinetic energy only remains approximately below 25 Joules for both the shoulder and elbow (14).

The definition of the catch-up phenomenon dictates that compensation for the loss of kinetic energy caused proximal in the linked-segment chain must occur in the successive segments to maintain pitching performance (12, 13, 16). According to the results of this study, some kinematic changes did occur, but this was observed in segments earlier in the kinetic chain and not in the expected successive segments following the pelvis and trunk (12–14). The peak angular velocity of the pelvis turned out to be significantly higher when pitchers had to throw fastballs with their impeded pelvis and trunk mobility. The trunk peak angular velocity did not show any differences between conditions, as well as the peak angular velocities and their separation times of the body segments of the throwing arm. Thus, assuming that the moment acting (from the pelvis) on the trunk remains unchanged, a higher pelvic peak angular velocity must lead to a higher peak power at the trunk. These findings indicate that pitchers are shifting more toward the use of the principle of optimal coordination of partial momenta, which appears to be sufficient to practically maintain pitching performance, as ball speed was found to be only somewhat lower in the impeded mobility condition. As a result of the applied circularly short-stretchable tape, the shorter separation time and higher pelvic peak angular velocity might indicate that pitchers are more likely to shift (partially) toward using the principle of optimal coordination of partial momenta and are therefore less dependent of using the summation of speed principle and employing a mechanism such as the “catch-up” phenomenon to maintain performance. This suggests that pitchers, presumably unconsciously, have the ability to shift (partially) more toward using one of the two principles or using them combined depending on the segments that form a link in the chain to maintain their pitching performance.

The pelvic and trunk mobility impediment, demonstrated by the shorter separation time, and the principle underlying fastball pitching did not appear to affect the elbow and shoulder moments. The results of this study showed that the elbow and shoulder moments were slightly lower when the pitchers' core segments mobility was impeded, although these differences were not statistically significant. This finding suggests that shifting (partially) more toward using one or both of the two principles does not appear to be at the expense of elbow and shoulder loading, meaning that the elbow and shoulder may not be at increased risk of developing (overuse) injuries.

While this study provides some valuable insights into the most well-known biomechanical principles to explain baseball pitching performance, it is important to note that limitations do exist. Firstly, the mobility impediment of the pelvic-lumbar link by the circularly short-stretchable tape was used to complicate the ability of the pelvis and trunk to rotate independently, which consequently should affect the time interval between the peak angular velocity of the pelvis and trunk (e.g., separation time). In this study, the applied circular short-stretch tape was able to successfully shorten the separation time and thereby the ball speed, which is consistent with the demonstrated positive association between the separation time and ball speed in the study of van der Graaff et al. (6). The method might mimic the effect of performance limiting factors such as pain, muscle fatigue, decreased muscle strength or neuromuscular control on pelvis and trunk movements (36, 37). However, the question remains to what extent the method used in this study approximates the actual effects of these factors on pelvis and trunk movement during fastball pitching. These performance limiting factors, which may be present when suffering from an injury or disability, could potentially reduce the mobility of these core segments as a protective mechanism to prevent, for instance, increasing pain. However, it does not appear to apply to factor muscle fatigue, as a simulated game study showed that the pelvis and trunk kinematics of collegiate baseball pitchers who pitched between 105 and 135 balls, approaching muscle fatigue, remained remarkably consistent (38). How these other performance limiting factors, besides muscle fatigue, affect the pelvic-lumbar link and thus baseball pitching as a whole should be explored in future studies. Secondly, the findings of this study showed that pitchers were unable to maintain their pitching performance and showed that any compensatory strategies did not manifest themselves clearly. The reason for this may be that pitchers not have been sufficiently stimulated or motivated to maintain their performances, simply because they were unaware of their pitching performance. The pitchers have not received direct feedback on their performance after each fastball pitched, nor have they been given time to practice in this impeded pelvic-lumbar mobility state. Perhaps if the pitchers had been exposed to direct feedback on their ball speeds after each fastball pitched in conjunction with a practice session, other compensation strategies might have emerged. Future research on compensatory mechanisms should consider incentivizing pitchers to maximize and maintain their performance through feedback on their current performance for potential compensatory mechanisms to manifest themselves. Moreover, general limitations such as fastball pitching in a controlled movement laboratory, on a custom-made pitching mound, throwing outwards from an indoor environment equipped with artificial grass and being rigged with reflective markers may have affected the pitchers' performance, which limits the generalizability of the findings in this study. Furthermore, despite the large number of pitches analyzed, the small sample size must be taken into account when interpreting the results of this study. However, data collection in this setting was necessary to obtain the most accurate biomechanical information possible from the baseball pitchers.

## Conclusion

In elite adolescent baseball pitchers, experimentally impeding pelvic and trunk mobility decreases ball speed when pitching fastballs, but does not affect peak joint power and mechanical loading of the elbow and shoulder. The higher pelvic peak angular velocity and the shorter separation time between the peak angular velocities of the pelvis and trunk indicates an absence of the hypothesized “catch-up” phenomenon following the summation of speed principle and a presence/manifestation of the principle of optimal coordination or partial momenta to practically maintain their performance.

## Data availability statement

The datasets presented in this study can be found in online repositories. The names of the repository/repositories and accession number(s) can be found below: 10.5281/zenodo.7022794.

## Ethics statement

The studies involving human participants were reviewed and approved by Scientific and Ethical Review Board (VCWE) of the Faculty of Behavior & Movement Sciences, Vrije Universiteit Amsterdam. Written informed consent to participate in this study was provided by the participants' legal guardian/next of kin.

## Author contributions

AL, MH, and HV contributed to the hypothesis formulation and design of this study. AL and BT contributed to the data acquisition and analysis. AL, BT, MH, and HV contributed to the interpretation of the data. AL completed the first draft of the manuscript. All authors contributed to the article and approved the submitted version.

## Funding

This work was supported by the Netherlands Organization for Scientific Research (NWO) Domain Applied and Engineering Sciences (AES, previously Technology Foundation STW) under grant agreement number (P16-28) and project number (R/003635). This NWO-funded project, called Breaking the High Load—Bad Coordination Multiplier in Overhead Sports Injuries part of the Citius Altius Sanius perspective program (Project 7), is a cooperative effort between the Royal Dutch Baseball and Softball Federation, Royal Dutch Lawn Tennis Federation, Vrije Universiteit Amsterdam, Delft University of Technology, Milé Fysiotherapy, PitchPerfect, and PLUX.

## Conflict of interest

The authors declare that the research was conducted in the absence of any commercial or financial relationships that could be construed as a potential conflict of interest.

## Publisher's note

All claims expressed in this article are solely those of the authors and do not necessarily represent those of their affiliated organizations, or those of the publisher, the editors and the reviewers. Any product that may be evaluated in this article, or claim that may be made by its manufacturer, is not guaranteed or endorsed by the publisher.
